# Metabolic Endotoxemia, Feeding Studies and the Use of the Limulus Amebocyte (LAL) Assay; Is It Fit for Purpose?

**DOI:** 10.3390/diagnostics10060428

**Published:** 2020-06-24

**Authors:** Karma Pearce, Dianne Estanislao, Sinan Fareed, Kelton Tremellen

**Affiliations:** 1 Division of Health Sciences, School of Pharmacy and Medical Sciences & Alliance for Research in Exercise Nutrition and Activity (ARENA), University of South Australia, Adelaide SA 5001, Australia; estdy001@mymail.unisa.edu.au (D.E.); Farsy029@mymail.unisa.edu.au (S.F.); 2 Department of Obstetrics Gynaecology and Reproductive Medicine, Flinders University, Bedford Park SA 5042, Australia; KTremellen@repromed.com; 3 Repromed IVF Adelaide, 180 Fullarton Road, Dulwich SA 5065, Australia

**Keywords:** metabolic endotoxemia, endotoxins, lipopolysaccharides, limulus amebocyte (LAL) assay, lipopolysaccharide binding protein (LBP) assay, adiposity, inflammation

## Abstract

The Limulus amebocyte assay (LAL) is increasingly used to quantify metabolic endotoxemia (ME), particularly in feeding studies. However, the assay was not intended to assess plasma at levels typically seen in ME. We aimed to optimize and validate the LAL assay under a range of pre-treatment conditions against the well-established lipopolysaccharide binding protein assay (LBP). Fifteen healthy overweight and obese males aged 28.8 ± 9.1years provided plasma. The LAL assay employed a range of pre-treatments; 70 °C for 15 and 30 min and 80 °C for 15 and 30 min, ultrasonication (70 °C for 10 min and then 40 °C for 10 min), and dilution (1:50, 1:75, 1:100, and 1:200 parts) or diluted using 0.5% pyrosperse. Seventeen different plasma pre-treatment methods employed prior to the use of the LAL analytical technique failed to show any relationships with either LBP, or body mass index (BMI; obesity), the biological trigger for ME (*p* > 0.05 for all). As expected, BMI positively correlated with LBP (r = 0.523, *p* = 0.052. No relationships were observed between LAL with any of the sample pre-treatments and LBP or BMI. In its current form, the LAL assay is unsuitable for detecting levels of endotoxin typically seen in ME.

## 1. Introduction

Endotoxin, also known as lipopolysaccharide (LPS) is derived from the Gram-negative bacterial outer membrane and consists of three key components; the O antigen, core oligosaccharide, and the lipid-A molecule [1]. These components may facilitate potent activation of innate immunity when triggered by low-grade endotoxemia, as demonstrated by the lipid-A molecule’s interaction with the toll-like receptor 4 (TLR4), mediating expression of pro-inflammatory cytokines [1]. The potentiation of a pro-inflammatory state via low presence of endotoxins may progress to the onset of inflammatory diseases [2,3]. The precise levels of endotoxin capable of damaging human health remains unclear, however modestly raised levels of endotoxins in human circulation have been increasingly linked in numerous studies to poor health and diseases such as; cardiovascular disease and atherosclerosis [3,4], insulin resistance and type 2 diabetes [2], and non-alcoholic fatty liver disease [5].

Currently, there are three very different analytical techniques to assess human endotoxin levels; the Limulus amebocyte assay (LAL assay), the lipopolysaccharide binding protein (LBP) assay and the endotoxin activity assay (EAA™), which we have previously demonstrated is not suitable for use under conditions of metabolic endotoxemia (ME) [5]. The LBPenzyme-linked immunosorbent assay (ELISA) assay provides an indirect measurement of sub-acute endotoxin which has been designed specifically for biological samples and measures an acute-phase reactant protein, predominantly produced by the liver, in response to endotoxin exposure [6]. The biological role of LBP is to deliver endotoxin to a co-receptor CD14, facilitating an interaction between endotoxin and TLR4, triggering a signaling cascade. This ultimately results in up-regulation and expression of pro-inflammatory cytokines [7,8]. The LBP has been used extensively to detect low levels of ME associated with obesity (body mass index; BMI) [9,10,11,12,13]. LBP in serum has also been used to detect endotoxemia in inflammatory bowel conditions, pancreatitis, cirrhosis, and sepsis (reviewed in [14]). However, the use of an indirect measurement may not be ideal for analyzing rapid changes in LPS exposure, such as postprandial endotoxemia, as the LBP response to endotoxin exposure is delayed and the accuracy of the test is dependent on normal hepatic function for production of the measured binding protein [15].

In contrast, there are three LAL assays which offer a direct measurement of endotoxin through initiating a blood clotting cascade, as Limulus polyphemus clots with Gram-negative bacteria during infection [16]. The most commonly used LAL assay is a chromogenic test. Briefly, the conversion of a pro-enzyme to its active form is catalyzed by endotoxin, which then splits p-nitroaniline (pNA) from a colorless substrate, which is quantified by the time taken for the optical density (OD) to increase by 0.2 OD at 405 nm and is quantitatively proportional to the concentration of endotoxin in the sample (Lonza, Walkersville, MD, USA). The LAL assay’s ability to assess endotoxin under conditions of sepsis is undisputed, however the manufacturer cautions that the assay was “not meant for testing blood or blood products, nor are they meant to diagnose, treat, or mitigate any disease or condition such as endotoxemia in man or animals” [17]. This is because inhibitors of the LAL assay present in complex biological samples, such as blood, interfere with endotoxin identification, particularly at low levels. Despite this, a number of research groups have quantified acute short-term changes in plasma endotoxin in healthy individuals before and after the ingestion of a fatty meal using the LAL assay [18,19,20,21,22,23,24,25]. However, none of these research groups have verified the accuracy of the determinations under postprandial ME. Additionally, there is a lack of consistency in the methodology employed under conditions of ME, namely in the variable dilution rates and heat treatments required for adequate inhibition of chemical interferences such as β-glucan [26], or protein modification in blood [27,28]. Therefore, with significant research interest on the effect of low levels of endotoxin exposure on health [29], there is a need to establish a gold standard assay to directly test for ME, thereby advancing research the field.

The aim of this study was to evaluate whether the LAL chromogenic assay was suitable to detect endotoxins under conditions of ME and if so, to then optimize the conditions of use and validate it against BMI, an established marker of ME and an established assay under conditions of fasting. This would provide a validated direct measure of ME which could be used to evaluate postprandial changes in ME during feeding studies.

## 2. Materials and Methods

### 2.1. Participant Recruitment

Men aged 18 to 50 years with a BMI > 25 kg/m² were recruited as part of a larger study (South Australian Medical Research Institute (SAMRI), Adelaide, South Australia) over a 3-month period in 2018. This overweight group was targeted as they have been reported to more likely exhibit ME [30,31,32], thereby having detectable levels of LPS in plasma. Men with an inflammatory or infective disease and consuming immunosuppressive medication (e.g., nonsteroidal anti-inflammatory drugs (NSAID), corticosteroids, or fish oil) or supplements that may alter intestinal function (e.g., probiotics, antibiotics in the last month) were excluded. The study was approved by the University of South Australia Human Research Ethics Committee in December 2018 (approval number: 200913).

### 2.2. Anthropometric Measurements

Anthropometric measures were collected. Height was measured to the nearest 1 cm (Seca, 216, Germany). Weight was measured using a bio-electrical impedance scale (Tanita, UM051, Tanita Corporation of America Inc. Arlington Heights, IL, USA). BMI was calculated using the equation body weight (kg)/height (m^2^) and classified using the World Health Organization (WHO) ranges; underweight (<18.50 kg/m^2^), normal weight (18.50–24.99 kg/m^2^), overweight (25.00–29.99 kg/m^2^) and obese (>30 kg/m^2^) [33].

### 2.3. Blood Collection

A trained phlebotomist collected venous blood samples into a plastic serum vacuette tube (9 mL, cat no: Lithium Heparin; Greiner, Kremsmünster, **Austria**), which we had shown to be free of endotoxin. All samples were allowed to clot for 2 h at room temperature and subsequently centrifuged at 3500 *g* at 4 °C for 15 min (Hettich Universal 320R, Geldermalsen, Netherlands). Raw plasma was decanted into pyrogen free tubes (cat: 80-507 USP Type 1 flint borosilicate glass tubes with caps; Lonza, Walkersville, MD, USA) using pyrogen free pipette tips (Cat; BE10051, Lonza, Walkersville, MD, USA). Aliquots of raw serum were stored at −80 °C [32] pending further analysis.

### 2.4. Assessment of Metabolic Endotoxemia

#### 2.4.1. Limulus Amebocyte (LAL) Assay 

##### Preparation of Standards

To prepare standards, a control standard endotoxin (CSE) bottle (Lonza Group Ltd., Walkersville, MD, USA) was reconstituted with LAL reagent water (LRW; lot no. 0000638444, exp: 21/06/2019; Lonza, Walkersville, MD, USA) to make a 50 EU/mL solution. Serial dilutions were prepared in pyrogen free glass tubes (Cat: N207; Lonza, USA; 5, 0.5, 0.05, 0.005, 0.003, 0.002, and 0.001 EU/mL) according to Supplementary Table S1.

##### Preparations of Plasma Samples

To inactivate endotoxin-neutralizing agents that inhibit endotoxin activity in the assay, a heat treatment and dilution protocol was employed. Samples were thawed to room temperature and a dry block heater (Ratek, Victoria, Australia) was pre-heated to 70 °C or 80 °C. For 1:10 pre-dilution, pyrogen-free glass tubes (Cat: N207; Lonza, USA) and pyrogen-free pipette tips (Cat: BE10051; Lonza, Walkersville, MD, USA) were used to aliquot 50 µL of sample into 450 µL of LAL reagent water (LRW; Lonza, Walkersville, MD, USA). Pre-dilution tubes were incubated at 70 °C for 15 and 30 min and 80 °C for 15 and 30 min per sample and then plunged into ice to cool to room temperature. Once cooled to room temperature, serial dilutions (1:50, 1:75, 1:100, and 1:200) were prepared as per Supplementary Table S2.

##### Preparation of Plasma Samples; Adaption for Ultrasonication

All samples were thawed and diluted 1 part in 10 with LRW. Samples were heated at 70 °C for 10 min and then subjected to an ultrasonic bath at 40 °C for 10 min (Digital Pulse Swept Power operating at 43 kHz ± 2 kHz sweep bandwidth with 20 Hz pulses; Soniclean, Transtek system, South Australia, Australia) and vortexed for 1 min to enhance the recovery of endotoxin.

##### Preparation of Plasma Samples; Adaption for Addition of Pyrosperse

All samples were thawed and diluted 1 part in 10 with LRW in pyrogen-free glass tubes (Cat: N207; Lonza, Walkersville, MD, USA) using pyrogen-free pipette tips (Cat: BE10051; Lonza, Walkersville, MD, USA). Pre-dilution tubes were incubated at 80 °C for 30 min per sample and then plunged into ice to cool to room temperature. Once cooled to room temperature, serial dilutions were prepared with 0.5% pyrosperse to minimize interferences in the reaction (inhibition or enhancement) [30] as per Supplementary Table S2.

##### LAL Analysis

Kinetic-QCL™ LAL kits from a single batch were used in the study (lot TL067Y5MJJ, exp:7/6/2020). The assay also used a Lonza micro-plate reader (ELX808LBS) which was calibrated by a Lonza representative at 405 nm (R = 0.999, slope = 1.656, y intercept = −0.068). One hundred microliters of each sample or standard were pipetted into a 96-well micro-plate (Cat no: 25-340; Lonza Walkersville, MD, USA) in duplicate. In addition, a further aliquot of 100 µL of sample, then a further 10 µL of 5 EU/mL standard in the same well was prepared in duplicate to create a 0.5 EU/mL spike in into the sample. This formed the positive product control (PPC). The plate was incubated in a micro-plate reader for 10 min at 37 °C. One hundred microliters of freshly prepared LAL reagent was added to each well. The plate was shaken for 30 s and the OD of each sample was read at 405 nm immediately after shaking and every 150 s thereafter for a period of 100 min with a Delta mean optical density (mOD) of 200. The data was processed using WinKQCL^TM^ 5 software (Lonza, Walkersville, MD, USA). A second instrument was also used in the analysis (Enspire, Perkin Elmer, Waltham, MA with accompanying software, USA) under the following conditions: incubation in a micro-plate reader for 10 min at 37 °C. One hundred microliters of freshly prepared LAL reagent was added to each well. The plate was shaken for 30 s and the OD of each sample was read at 405 nm immediately after shaking and every 40 s thereafter for a total of 200 reading (133 min). The reaction time was defined, as recommended by the manufacturer, as the time taken for OD to increase by 0.2 units from the initial (baseline) OD value of either standards or samples (Lonza, Walkersville, MD, USA). Polynomial models were generated in Microsoft Excel 2010 based on the reaction times in seconds of a series of endotoxin concentrations between 0.001 and 50 EU/mL in LRW.

##### Summary of Polynomial Model Validation and Quality Measures

**Figure 1:**
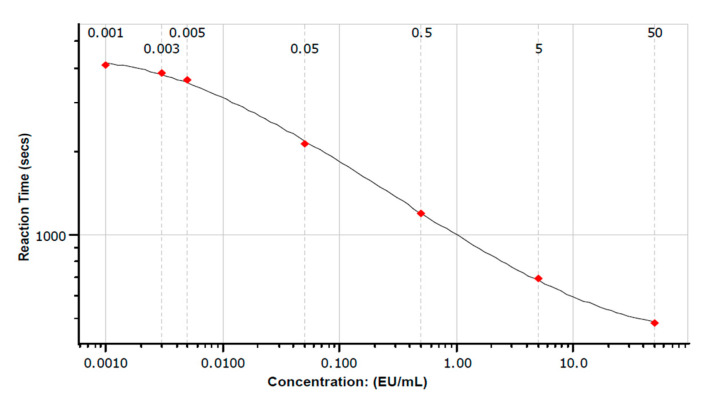
Polynomial model of reaction time vs. endotoxin concentration. Figure shows polynomial standard curve fit: For the fitted log-log linear model r = −0.996, slope = −0.214, y intercept = 3.026, coefficient of variation (CV%) = 2.02%.

The coefficient of correlation (r) was ≥0.980 for all polynomial models. Reproducibility was determined by percent coefficient of variation (%C.V.) of reaction times for replicates for all samples and standard solutions as ≤10%. To be included in the analysis, % recovery of positive product controls (%PPC) were between 50–200% as per methodology (Lonza, Walkersville, MD, USA). All blank controls were less than the minimum absorption of the standards and samples. This enabled a polynomial model to be utilized to produce a polynomial model and allow prediction of endotoxin concentrations in individuals with ME (see Figure 1 for example). The limit of detection (LOD) for all samples was calculated as the minimum standard concentration multiplied by sample dilution level; 1:50 dilution = 0.05 EU/mL, 1:75 = 0.075 EU/mL, 1:100 = 0.1 EU/mL, and 1:200 = 0.2 EU/mL.

#### 2.4.2. Lipopolysaccharide Binding Protein (LBP) Endotoxin Measurement

Metabolic endotoxemia was quantified indirectly by LBP analysis using an ELISA system according to the manufacturer’s guidelines (Hycult, Uden, Netherlands) as previously published by our group [34,35] with the minimum detectable concentration of LBP being 4.4 ng/mL. LBP was determined at 450 nm (using a Multiskan Ascent 96/384 Plate Reader, Oitti, Finland). The data was downloaded in Excel and analyzed using elisaanalysis.com where the OD values of the standards were plotted against logarithmic transformed concentrations of LPS in the standards. The concentration of LPS in each sample was determined by reference to the polynomial model.

### 2.5. Data Interpretation

Based on previous work, in which we observed a significant correlation between BMI and LBP in 10 overweight and obese men (r = 0.649, *p* = 0.042, CI_95_ 0.028–1.269, [34]), we believed we required a sample size of 15 overweight and obese men allowing for dropouts to observe a relationship between LBP and BMI in the current study. Statistical analyses were conducted using International business machines corporation (IBM) Statistical Product and Service Solution software, version 24 (SPSS Inc., Chicago, IL, USA). All variables were reported as mean ± SD where normally distributed, or as median ± inter quartile range when not normally distributed using the Shapiro–Wilk test. Homogeneity was evaluated using Levene’s test of equality. Correlations were assessed using the Pearson method. A two-way ANOVA was employed to determine statistical differences between methods of analysis and sample pre-treatments to determine endotoxin levels. Post-hoc analysis was performed using Tukey analysis. Significance was set at *p* < 0.05.

## 3. Results

### 3.1. Participant Demographics

Fifteen healthy males were recruited to this study. The mean ± SD age and BMI was 28.8 ± 9.1 years and 29.6 ± 4.5 kg/m² respectively. From the cohort, there were 73.3% overweight and 26.7% obese. The men were non-smokers, were not on special diets and consumed less than four standard drinks per day.

### 3.2. LAL Analysis

#### 3.2.1. Summary of Polynomial Model Validation and Quality Measures

The coefficient of correlation (r) was ≥0.980 for all polynomial models and agreement between replicates (%CV) was <10% as per methodology. Figure 1.

#### 3.2.2. Sample Heat Treatment, Deactivation Time and Dilution on Serum Endotoxin Levels

**Figure 2:**
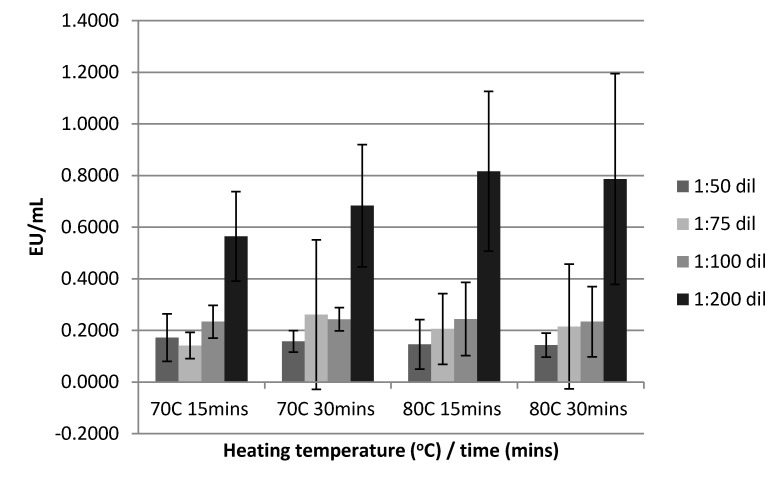
The effect of heat treatment, deactivation time, and dilution on serum endotoxin levels analyzed by the limulus amebocyte lysatehttps://en.wikipedia.org/wiki/Limulus_amebocyte_lysate (LAL) assay.

The percentage of samples meeting the inclusion criteria for data analysis (%CV < 10%, %PPC 50–200%) are outlined in Table 1. The use of acid treatment was deemed unnecessary as the pH of all samples after heat treatment was below a value of 8. Sample dilution only meaningfully and significantly impacted on the detected endotoxin levels when the samples were diluted to 1:200; the magnitude of the difference in detectable endotoxin levels varied between 228% to 298% for samples diluted 1:200 compared to other dilutions (1:50, 1:75, and 1:100) which had similar heat deactivation conditions. Samples heat treated at 70 °C for 15 min and diluted to 1:200 significantly differed from samples diluted 1:50, 1:75, and 1:100 (*p* = 0.003, *p* < 0.001, and *p* = 0.006, respectively), or samples heat treated at 70 °C for 30 min at a dilution of 1:50, 1:75, and 1:100 (*p* = 0.004, *p* = 0.023, and *p* = 0.008, respectively), or samples heat treated at 80 °C for 15 min at a dilution of 1:50, 1:75, and 1:100 (*p* = 0.003, *p* = 0.016, and *p* = 0.009, respectively) or samples heat treated at 80 °C for 30 min at a dilution of 1:50, 1:75, and 1:100 (*p* = 0.001, *p* = 0.003, and *p* = 0.003, respectively). The effect of sample pre-treatment with heat and time on dilution is displayed in Figure 2.

#### 3.2.3. LAL Adaption for Ultrasonication

The coefficient of variation of the reaction time between replicates for the polynomial model was 3.5% (0–6.1%), with r = 0.997. Unfortunately, only seven samples exhibited %PPC, with a %CV for duplicate analysis of the positive product control sample <10% (% PCC (31–604%), CV (0.5–60%)). Of these seven samples, only five samples met inclusion criteria of %CV for the duplicate sample analysis, suggesting ultrasonication in combination with a 10-fold dilution was not a robust method to detect endotoxin levels in serum.

#### 3.2.4. LAL Adaption for Addition of Pyrosperse

The coefficient of variation of the reaction time between replicates for the polynomial model was 2.8% (0–3.9%), with r = 0.998. The addition of 0.5% pyrosperse made no significant difference to the levels of endotoxin detected for serum samples diluted 1:200 and heated to 80 °C for 30 min compared to samples without the addition of pyrosperse (*p* = 0.374).

#### 3.2.5. Relationship between BMI and Endotoxemia

The relationship with LBP detected metabolic endotoxemia and BMI was significant (r = 0.523, *p* = 0.052, CI_95_ 0.004–1.051). However, there were no relationships between BMI and LAL assayed endotoxemia irrespective of heat treatment time, temperature, dilution, or the use of ultrasonication.

#### 3.2.6. Comparison of LBP to LAL

**Figure 3:**
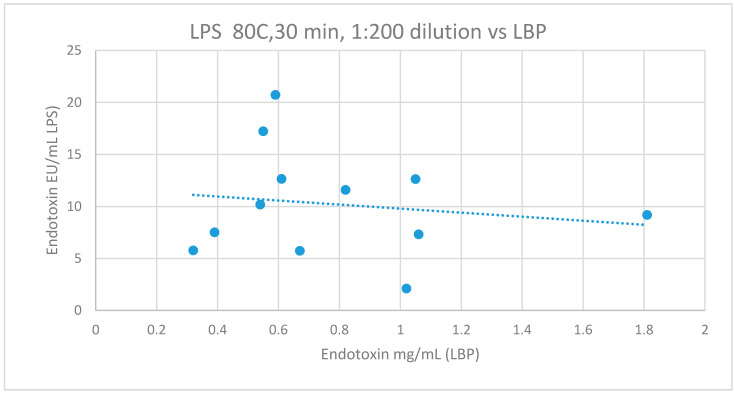
The relationship between LPS (with sample pre-treatment of 80 °C, 30 mins, 1:200 dilution) and LBP. Lipopolysaccharide (LPS), Lipopolysaccharide binding protein (LBP), r = −0.278, *p* = 0.381, CI_95_ (−0.916–0.381).

Metabolic endotoxemia was quantified indirectly by LBP analysis; 10.2 ± 5.2 ug/mL. There were no significant correlations between LBP and samples heat treated at 80 °C for 15 min or 30 min after dilution to either 1:100 (r = −0.578, *p* = 0.134, CI_95_ = −1.393–0.238; r = −0.493, *p* = 0.102, CI_95_ = −1.881–0.087) or 1:200 for 15 min or 30 min and analyzed using the LAL method of analysis (r = −0.078, *p* = 0.841, CI_95_ = −0.208–0.991; r = −0.278, *p* = 0.381, CI_95_ = −0.916–0.381; Figure 3). Additionally, there were no significant correlations with LBP measures of endotoxin and any other sample pre-treatment and LAL measures of endotoxin (LPS).

## 4. Discussion

Metabolic endotoxemia is increasingly reported in the literature, yet there is no gold standard method of analysis for low levels of circulating endotoxins. Human research studies using the LAL assay often poorly report the methodology employed; sample pre-treatment conditions are often not stated [36], referenced to manufacturer’s instructions (which are non-existent for endotoxin levels observed under conditions of ME using a Lonza kit) [37], or are scant [18,30].

The key finding of this study was that existing published LAL sample inhibitor neutralization pre-treatments of 70 °C and 80 °C for both 15 and 30 min, vortexed for 2 min and subsequently diluted 1:50, 1:75, 1:100, and 1:200, or diluted 1:10, heat treated to 70 °C for 10 min, vortexed for one minute and ultrasonicated for 10 min or diluted using 0.5% pyrosperse and analyzed using the LAL chromogenic assay were not suitable to detect endotoxin levels in plasma, amongst overweight men typically exhibiting ME. There are two key findings which substantiate this. Firstly, ME has long been associated with obesity. In the current study, obesity (BMI) was significantly related to the indirect measure of endotoxemia (LBP) with a moderate effect size, yet there were no relationships between BMI and the direct measure of endotoxemia (LPS) using the LAL chromogenic assay under any of the sample pre-treatment conditions examined. The positive correlation between BMI and LBP concur with many other research groups [9,35,38], including our own [34], and in varied ethnic populations [11,12], yet surprisingly many research groups reporting on ME measures, particularly those addressing methodology, fail to report on this relationship.

Secondly, the literature commonly reports both the LAL chromogenic assay [21,30,31] and LBP ELISA assay for quantifying ME [21,32], despite the mechanisms for detection being very different [7,16]. As such, one would expect to observe a direct positive relationship between both measures in the fasting state. However, this was not the case in our study. Endotoxin levels determined using the LBP ELISA assay failed to correlate with any of the 17 variants of sample pre-treatment for analysis performed using the LAL assay. Interestingly, we have previously failed to find a relationship between LBP and LPS in 10 healthy overweight and obese males in the fasting state (r = −0.341, *p* = 0.369; unpublished data) and Ghamim et al. who used both LAL and LBP assays to measure ME failed to detect a fasting relationship between the two measures of endotoxemia [21].

There are several possible explanations for this lack of correlation; the most likely was deactivation of endogenous sample compounds capable of interfering with the clotting cascade was incomplete [39,40]. The most common methods reported for ameliorating this inhibition or enhancement have been the combination of dilution and heating [25,41,42], solvent extraction [39], sonication [25,43], and acid treatment [41,44], or combinations thereof. While the most commonly reported heat treatments, dilutions, and the use of sonication were employed in this study, the lack of correlation between LBP and LPS suggests that they were not sufficient to deactivate the inhibitors. Secondly, LBP is produced principally by the liver in response to LPS exposure, making LBP levels dependent on hepatic function, unlike the LAL assay which is a direct measure of LPS activity. In an acute exposure to a bolus of LPS, LBP is consumed by binding to LPS and transported to responding immune cells-producing a paradoxical fall in LBP rather than increase. However, as all of the men in this study were heathy, not experiencing any acute event likely to produce a bolus exposure to LPS, this pattern is unlikely to explain the lack of correlation between LBP and LAL quantified endotoxemia.

Finally, ME mirrors the activity of LPS, which in the case of the LAL analysis is based on the structure of the lipid-A moiety and polysaccharide chain [45]. We acknowledge that various LPS species exist with numerous activities according to their molecular structure, so it is possible that the increased levels of endotoxin may be the result of an increased number of LPS molecules, or a similar level of LPS molecules with increased activity due to different chemical structures [31]. It has also been suggested that this discrepancy could be more fully understood by assessing the biochemical forms of LPS in parallel with the LAL assay at an optimum dilution [31]. However, while this has been successful under conditions of sepsis [46], correlations for LPS determined by the LAL analysis and biochemical methods of analysis (HPLC-MS/MS) are still lacking under conditions of ME [47].

The strengths of this study include the quality measures taken to ensure the accuracy of the polynomial model; all correlation coefficients were greater than 0.980, and the agreement between replicates (%CV) was less than 10%. The activation of the LAL test is also easily detected by a fast response in the positive product control (PPC) well. The manufacturer specifies the reactivity of such PPC should lie within 50–200% and we report on compliance to this range and only included samples adhering to this range in the analysis. Additionally, the percentage coefficient of variation between two replicate analyses of the same sample was less than 10% as recommended by the manufacturer. Care was also taken to minimize cross contamination through the use of endotoxin free reagents (LAL water), pipette tips, glass storage containers, etc. [32], and this was confirmed through the use of ‘reagent blanks’. The type of collection tube has been shown to affect the analysis; in this study, blood was collected in heparin tubes, centrifuged within 3 h of collection and then transferred to endotoxin free glass bottles and stored at −80 °C pending analysis. While others have reported heparin tubes to increase endotoxin levels [48,49] and hydrophobic polymers to bind LPS [50], in this case, limited storage for 3 h was not found to have an impact on the analysis with no detectable level above the limit of detection.

We acknowledge several potential weaknesses in our study. Laugerette et al. suggested sonication would disperse the aggregates of LPS into smaller and more uniform particles to enhance detection [31]. The lack of correlation between this measure of LPS and LBP suggest the conditions in our study were insufficient to completely reduce this aggregation; (Digital Pulse Swept Power operating at 43 kHz ± 2 kHz sweep bandwidth with 20 Hz pulses). The study also failed to control for the possible presence of proteases and glucans; it is possible that proteases [50,51] or glucans [26], if present in sufficient quantities in the blood, could have inhibited the clotting cascade in the LAL assay. The recovery of LPS from serum after it has bound to lipoproteins is also challenging. While some suggest that re-extraction of plasma using chloroform could be used to release LPS from inhibitory proteins and lipoproteins [52], others report as little as ~0.001% of the spiked biological activity is recovered using this method [53], suggesting that even after additional preparative work, the majority of LPS in the circulation residing in lipoproteins was not detectable by the LAL assay.

## 5. Conclusions

The LAL method of analysis is often used and importantly reported in peer reviewed literature as a measure to detect endotoxin levels under conditions of ME, despite the manufacturer not endorsing its use under these conditions. However, 17 different pre-treatment methods employed prior to the use of the LAL chromogenic assay failed to show any relationships with BMI or LBP suggesting in its current form, it is unsuitable for use for detecting levels of endotoxin typically seen in ME. LBP is the best available surrogate marker of endotoxin exposure, but cannot be used for measuring acute changes in exposure over short periods of time (minutes/hours). Furthermore, we suggest future studies employing LAL chromogenic methodologies under conditions of ME report correlations with BMI and LBP (or an alternative method of analysis) as a quality measure.

As ME is increasingly emerging as a mediator in many chronic diseases, there is an urgent need for more research to establish a robust ‘gold standard’ analytical tool to determine endotoxin levels in the systemic system, particularly with feeding studies. There are a number of emerging analytical techniques involving biosensors [54], electron microscopy, dynamic light scattering (DLS), fluorescence resonance energy transfer (FRET), and docking programs in the endotoxin-protein analysis (reviewed in [53]) that may offer an alternative pending investigation under conditions of ME.

## Figures and Tables

**Table 1:** Percentage of samples meeting inclusion criteria for analysis using the LAL assay.

Sample Pre-Treatment													
		70 °C/15 Mins			70 °C/30 Mins			80 °C/15 Mins			80 °C/30 Mins		
		Sample	PPC		Sample	PPC		Sample	PPC		Sample	PPC	
Dilution		CV	Rec	CV	CV	Rec	CV	CV	Rec	CV	CV	Rec	CV
1:50	%	100	40	100	100	33	100	100	33	100	100	40	100
	range	(1–8)	(28–62)	(1–6)	(1–4)	(35–188)	(1–5)	(1–4)	(37–67)	(1–15)	(1–6)	(37–67)	(1–8)
1:75	%	100	46	100	100	46	100	100	47	100	100	53	100
	range	(1–9)	(24–69)	(1–7)	(1–4)	(35–62)	(1–4)	(1–10)	(26–83)	(2–9)	(1–4)	(37–67)	(1–6)
1:100	%	100	53	100	100	46	100	100	53	100	100	60	100
	range	(1–6)	(38–96)	(1–10)	(1–23)	(22–90)	(1–3)	(1–10)	(24–103)	(2–6)	(1–14)	(35–93)	(1–8)
1:200	%	100	46	100	100	46	100	100	100	100	100	87	100
	range	(1–3)	(28–72)	(1–4)	(1–4)	(32–110)	(1–5)	(1–5)	(50–131)	(2–7)	(1–7)	(19–103)	(1–8)

*n* = 15 healthy males. Rec; recovery. Samples were prepared in duplicate and their percentage coefficient of variation (%CV) reported. Additionally, spiked positive product controls (PPC) were prepared for each sample in duplicate. The recovery for the PPC and the %CV for the duplicate is reported.

## Supplementary Materials

The following are available online at https://www.mdpi.com/2075-4418/10/6/428/s1, Table S1: Dilution protocol for standards, Table S2: Dilution protocol for samples.
