# Supplementary material for: Metabolic Endotoxemia, Feeding Studies and the Use of the Limulus Amebocyte (LAL) Assay; Is It Fit for Purpose?
Source: Diagnostics (Basel). 2020 Jun 24;10(6):428. doi: 10.3390/diagnostics10060428 (PMC7345849; doi:10.3390/diagnostics10060428)
Supplement: Supplementary file 1 [file diagnostics-10-00428-s001.zip › diagnostics-815133-supplementary.docx]

Table S1. Dilution protocol for standards

| Endotoxin Concentration (EU/ml))) | Volume of LRW | Volume of Endotoxin Solution |
| --- | --- | --- |
| 5 | 0.9ml | 0.1ml of 50.0 EU/ml |
| 0.5 | 0.9ml | 0.1ml of 5.0 EU/ml |
| 0.05 | 0.9ml | 0.1ml of 0.5 EU/ml |
| 0.005 | 0.9ml | 0.1ml of 0.05 EU/ml |
| 0.003 | 0.4ml | 0.6ml of 0.005 EU/ml |
| 0.002 | 0.6ml | 0.4ml of 0.005 EU/ml |
| 0.001 | 0.8ml | 0.2ml of 0.005 EU/ml |

Dilution protocol for standards using endotoxin solution and LRW for LAL assay. Adapted from Lonza [17].

Table S2: Dilution protocol for samples

| **Dilution** | **Volume of LRW** | **Volume of Preceding Dilution Added to LRW** |
| --- | --- | --- |
| 1:50 | 800µl | 200µl of 1:10 pre-dilution |
| 1:75 | 425µl | 75µl of 1:10 pre-dilution |
| 1:100 | 500µl | 500µl of 1:50 dilution |
| 1:200 | 250µl | 250µl of 1:100 dilution |

Dilution protocol for samples using heat-treated sample and LRW for LAL assay. Adapted from Lonza [17].
